# Adjusting LCA Allocation Methods for Cement Industry: A Production-Based Approach to Energy Conservation and Emission Reduction

**DOI:** 10.3390/ma18112483

**Published:** 2025-05-25

**Authors:** Zhengze Li, Xuan Chen, Anming She, Xiaolu Guo, Chunxiang Qian

**Affiliations:** 1 Key Laboratory of Advanced Civil Engineering Materials of Ministry of Education, School of Materials Science and Engineering, Tongji University, Shanghai 201804, China; lizhengze@tongji.edu.cn (Z.L.);; 2 School of Materials Science and Engineering, Southeast University, Nanjing 211189, China; cxqian@seu.edu.cn; 3 Research Center for Green Construction Materials & Carbon Utilization, Southeast University, Nanjing 211189, China

**Keywords:** life cycle assessment, cement industry, allocation method, solid waste management, economic allocation, polluter pays principle

## Abstract

Life cycle assessment (LCA) is an excellent tool for developing energy saving and emission reduction strategies. However, inconsistencies in the summary calculation methods in LCA can significantly affect the reliability of LCA reports, such as the allocation of environmental loads related to solid waste. Essentially, allocation methods are used to allocate responsibility for environmental loads in situations where boundaries are unclear, and therefore, they are susceptible to regional, industry, and regulatory influences. For a long time, there has been controversy over the selection of allocation methods. This study is based on actual production data from a typical cement plant in South China. Multiple parallel LCA cases were carried out using different allocation methods, and different allocation methods were analyzed. Concepts such as driving force, active/passive environmental load, Valorized Solid Waste (VSW), and Non-Valorized Solid Waste (NVSW) were introduced. Analysis shows that the choice of allocation method directly affects the effectiveness of energy saving and emission reduction plans in the LCA report. For VSW, the economic allocation method has been proven to have high universality, effectively capturing the driving forces of economic factors. For NVSW, based on the “polluter pays principle” and active/passive environmental load, we introduced the Collaborative Disposal Allocation Method (CD method). In this study, the environmental benefits of domestic waste collaborative disposal were calculated using the CD method, resulting in a 2.25% reduction in global warming potential (GWP) and a 45.39% reduction in respiratory inorganics (RIs).

## 1. Introduction

With the intensifying global climate change, energy conservation and emission reduction have become critical issues that urgently need to be addressed. The Paris Agreement, signed in 2015, aims to limit global warming to below 1.5 °C [1]. This means that net-zero greenhouse gas emissions need to be achieved by 2050. To this end, governments are increasingly making climate change a national strategic priority. For example, China proposed the “dual carbon” strategic goal in 2020, aiming to achieve the carbon peak by 2030 and carbon neutrality by 2060 [2], in order to alleviate the global greenhouse effect.

Cement, as the most widely used building material in modern society, has always been one of the major sources of CO_2_ emissions. According to statistics, cement production accounts for approximately 5% of global carbon emissions [3]. Given that China’s cement production accounts for approximately half of the global production. The transformation of China’s cement industry towards energy conservation and emission reduction has significant practical significance. During the cement production process, CO_2_ is directly emitted from raw materials. In fact, most of the carbon emissions in cement production come from the decomposition and decarbonization of raw mineral materials and the combustion of fuels [4]. Therefore, energy conservation and emission reduction measures in the cement industry are often not directly feasible. Common strategies mainly include reducing cement consumption, improving resource utilization efficiency, and increasing the proportion of non-conventional raw materials [5,6,7,8,9,10]. Decision-makers usually need to select appropriate strategies based on their specific circumstances to achieve ideal environmental benefits. In this process, life cycle assessment (LCA) has become an excellent tool for formulating energy conservation and emission reduction strategies.

LCA is a systematic method that quantitatively calculates and conducts research on the entire life cycle of a product, from raw material acquisition to design, manufacturing, use, recycling, and final disposal. This method helps identify potential environmental impacts throughout the process and evaluates the environmental performance of the process. According to ISO 14,040 [11], LCA consists of four inter-related steps: Goal and Scope Definition (GSD), life cycle inventory (LCI), life cycle impact assessment (LCIA), and Life Cycle Interpretation.

In general, LCA studies include the entire life cycle “from cradle to grave” and are analyzed based on a Functional Unit (FU) [12], evaluating the complete life cycle of a product. However, for cement, its application scenarios are often diverse, such as in roadways, bridges, and adhesives. Therefore, it is impractical to define a unique and complete life cycle solely for cement as the research object. The analysis must conclude at an intermediate stage [13], namely “from cradle to gate”: the independent process from raw material extraction to the production of cement products.

Briefly, LCA research on cement involves the following steps: firstly, determining the research object of LCA, typically using the performance of cement as the FU; secondly, establishing the system boundary of the study, which includes the main processes of cement production and potential resource consumption; and thirdly, collecting life cycle data, including raw material consumption, energy consumption, and emissions. Once sufficient data are prepared, a summary calculation of environmental impacts is conducted [14,15,16]. Finally, the calculation results are analyzed to provide theoretical support for the formulation of sustainable development strategies in cement enterprises.

However, it should be noted that the results of LCA studies not only depend on life cycle data but are also closely related to the final summary calculation method. It is possible for misleading results to arise due to differences in LCA algorithms, as the industrial processes involved in cement production practice are much more complex than those in theory. Currently, there is no unified standard for LCA algorithms in the cement industry, and significant differences exist in LCA results using different accounting boundaries and calculation methods [17]. This may lead to a decrease in the reliability of LCA reports.

For example, using solid waste as alternative raw materials and fuels has been one of the common means to reduce the environmental load in cement production. Typically, fly ash, coal gangue, and steel slag powder can be used as alternative raw materials [18,19,20], while domestic waste and waste rubber can serve as alternative fuels [21,22]. In many previous LCA studies on cement production, solid waste was considered to have no upstream environmental load during accounting [23]. The use of solid waste as a raw material by cement plants is a process of disposing of solid waste, and the environmental load of the solid waste itself should be borne by the upstream production process that generates the solid waste [5]. However, the European Union’s Waste Framework Directive (WFD) [24] clearly states that if solid waste meets the following four conditions, it should be considered a by-product and required to bear a portion of the upstream environmental load:

(1) The application of this material is certain;
(2) The material is produced as an integral part;
(3) The application of the material only requires industrial practice without further processing;
(4) The use of the material is legitimate and meets the requirements of product, environmental, and health protection standards.

On this basis, the allocation of environmental loads from solid waste has become an important factor in assessing the fairness and reasonableness of LCA studies. Karina has summarized various viewpoints [25], and overall, the main controversy regarding the accounting of the environmental load of solid waste currently focuses on the lack of detailed upstream processes or the belief that solid waste has little impact on the total environmental load. According to the cut-off rules [11] that determine whether to ignore certain data, the environmental impact of a material can be ignored if its proportion is less than 1% or its a low-value material from upstream processes. However, the ignored portion should not exceed 5% of the total. Due to the specificity of cement production, which often involves the co-disposal of solid waste [26], the amount of alternative raw materials used in cement production can sometimes reach more than 10%. In such cases, the reasonable allocation of environmental loads from solid waste can significantly improve the reliability and fairness of LCA results for cement production.

Calculating the environmental load of solid waste requires the use of allocation methods. In short, the allocation method is an algorithm for determining the allocation of environmental responsibility for solid waste within the framework of LCA. At present, there are various theories about allocation methods, such as mass allocation, economic allocation [27], and system extension method (SE method) [28]. Due to the significant impact of allocation methods on the results of LCA research [24], and the fact that allocation methods themselves do not affect the overall environmental load of society, their purpose is to allocate responsibility for environmental load in situations where boundaries are blurred. Therefore, it is deeply influenced by regions, industries, and regulations. There has always been controversy over the fairness and rationality of the selection of allocation methods. This study uses actual production data from a typical cement plant in southern China, focusing on specific issues in specific cases. Multiple parallel LCA cases were conducted using various allocation methods. Based on objectivity, comprehensiveness, and practicality, a series of relatively universal allocation methods were selected to enhance the reference value of LCA results. This provides suggestions and data references for the localization of LCA algorithms and the unification of evaluation standards for the global cement industry.

## 2. Method

### 2.1. Selection of Allocation Methods

Currently, the commonly used allocation methods in LCA studies are mass allocation and economic allocation. These methods essentially follow the same allocation logic but differ in the choice of allocation coefficient. Their definitions and calculation methods are as follows [23,27]:

(1) $$F_{upper-summary}=F_{product}+m_{waste}\cdot F_{waste}$$

Here, *F_upper-summary_* represents the actual total environmental load of producing 1 unit of the main product (coal, steel, etc.) in the upstream process of solid waste. *F_product_* represents the environmental load that needs to be accounted for in the production of 1 unit of the main product. *m_waste_* represents the mass of solid waste generated per unit of the main product, which is determined by the production process of the upstream stage. *F_waste_*, on the other hand, represents the environmental load that needs to be accounted for per unit of solid waste (such as coal gangue or steel slag), and it is directly related to the choice of allocation coefficient.

The relationship between *F_product_* and *F_waste_* is directly determined by the allocation coefficient *C*, as follows:

(2) $$\frac{m_{waste}\cdot F_{waste}}{m_{waste}\cdot F_{waste}+F_{product}}=\frac{m_{waste}\cdot F_{waste}}{F_{upper-summary}}=C$$

The allocation coefficient *C* can be divided into the mass allocation coefficient *C_m_* and the economic allocation coefficient *C_e_*; their calculation formulas are as follows:

(3) $$C_{m}=\frac{m_{waste}}{1+m_{waste}}$$

(4) $$C_{e}=\frac{{\$}_{waste}\cdot m_{waste}}{{\$}_{product}+{\$}_{waste}\cdot m_{waste}}$$

where $ represents the economic value of 1 unit of the main product or solid waste. Therefore, when using solid waste as a raw material in cement production, the environmental load should be calculated as follows:

(5) $$F_{cement}=F_{cement-net}+\frac{m_{waste-process}}{m_{waste}}\cdot F_{upper-summary}\cdot C+m_{waste-process}\cdot F_{secondary-working}$$

In this formula, *F_cement_* represents the environmental load that needs to be accounted for in the production of 1 unit of cement. *F_cement-net_* represents the net environmental load of producing 1 unit of cement without considering the upstream environmental load of any solid waste. *F_secondary-working_* represents the environmental load generated during the processing of treating 1 unit of solid waste to meet the standards for participating in cement production, such as drying, grinding, and other processes. And *m_waste-process_* represents the mass of solid waste required to produce 1 unit of cement, which is determined by the cement production process.

Correspondingly, according to the traditional cut-off rules (CO method), it is believed that the environmental load of solid waste should be fully borne by the upstream process that generates the waste [5]. Therefore, the environmental load that needs to be accounted for in the production of 1 unit of cement using solid waste as a raw material would only be

(6) $$F_{cement}=F_{cement-net}+m_{waste-process}\cdot F_{secondary-working}$$

The SE method suggests that the use of solid waste in cement production avoids the use of some conventional raw materials, and the environmental load of these avoided raw materials should be subtracted from the upstream process of solid waste accounting [28,29,30]. The SE method is relatively more suitable for accounting for the upstream processes that generate solid waste and comparing different recycling methods [5]. However, cement production is typically in the downstream stage of the industrial process. Therefore, this study does not use the SE method for calculation but chooses to conduct a horizontal comparison between the CO method, mass allocation, and economic allocation.

### 2.2. Life Cycle Assessment

The current LCA model is established based on ISO 14,040 [11] and ISO 14,044 [31], with data sourced from actual production records of cooperating cement plants and supplemented by the local Chinese CLCD [32] database.

### 2.3. Functional Unit and System Boundary

The traditional choice of FU for LCA of cement or concrete has often been 1 t of cement or 1 m^3^ of concrete [33,34,35,36]. However, this may not always be accurate since 1 t of cement or 1 m^3^ of concrete mainly reflects the quantity or filling effect, ignoring one of the most crucial properties of building materials: strength [37]. For cement, as the proportion of solid waste in the raw materials increases, there may be a corresponding decrease in the components that provide strength, leading to a reduction in overall strength. This consideration is limited to the “from cradle to gate”, excluding the service scenario. If the cement is used in structural concrete, lower cement strength would require a higher amount of cement, significantly limiting environmental benefits. Therefore, determining the FU for LCA should consider the product’s performance. It is crucial to ensure that products from different production processes have the same performance when comparing the environmental impacts of these processes. In addition, according to the research objectives of LCA, FU may have multiple options, and choosing the most suitable FU can greatly improve the reliability and objectivity of LCA research.

**Figure 1:**
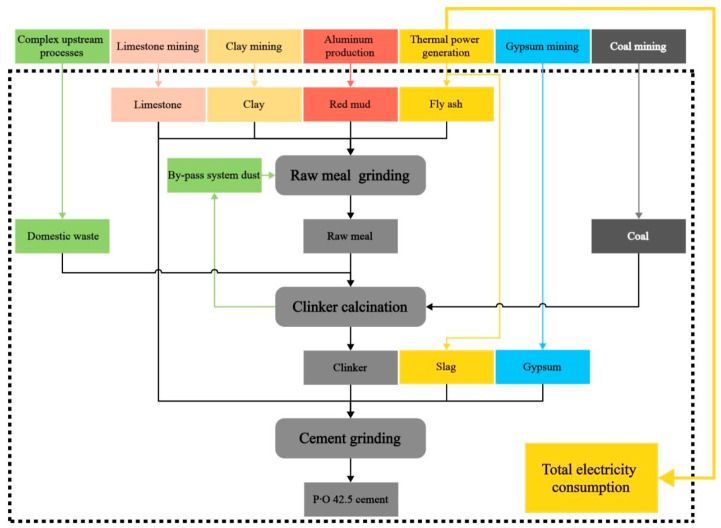
System boundary of P·O 42.5 cement.

In this series of LCAs, the only variable is the allocation method. The system boundary is constructed based on the same product from the same production line at the same time. Therefore, the selected FU is 1 t of P·O 42.5 (Chinese standard; it may belong to CEM II according to EU norms) ordinary Portland cement with a 28d strength of 55 MPa produced by the cooperative cement plant. Based on the above information and actual situation of the production line, the system boundary is constructed as Figure 1 shows.

It can be seen that besides conventional raw materials such as limestone and coal, the cement plant also uses solid wastes like red mud, fly ash, domestic waste, and slag in the production of P·O 42.5 cement. Among them, red mud and fly ash are used as alternative raw materials in the raw meal grinding process, domestic waste is used as an alternative fuel in the clinker calcination process, and slag is used as a mixing material in the grinding of P·O 42.5 cement.

### 2.4. Life Cycle Inventory

Life cycle inventory analysis is the collation and summary of all inputs and outputs within the system boundary. In this LCA, the consumption of various raw materials and energy sources was derived from actual production data from the cement plant. Pollutant emission data were obtained from real-time monitoring data from the cement plant’s numerical control center and third-party verification reports. Pollutant emission data not included in the monitoring scope were sourced from the CLCD [32] database. Upstream process data for conventional raw materials, solid waste, and energy were obtained from the CLCD [32] database and the related literature [38,39].

As shown in Figure 1, the cement plant’s P·O 42.5 cement production line can be divided into three sections: raw meal grinding, clinker calcination, and cement grinding. For ease of calculation, this study will use 1 t of raw meal, 1 t of clinker, and 1 t of cement as nodes to convert the life cycle inventory, presenting a tree-like data structure as shown in Table 1.

Transportation data are estimated based on the geographical location of various raw material suppliers and the cement plant, as shown in Table 2.

Next, regarding the selection of allocation methods, the solid wastes within the system boundary of this LCA include red mud, fly ash, domestic waste, and slag. Among them, red mud, fly ash, and slag meet the conditions of the WFD and should be considered as by-products. An allocation method is required to calculate their upstream environmental load.

Firstly, regarding red mud, which is a waste product of alumina production, research indicates that the production of 1 t of alumina generates between 1 and 1.5 t of red mud [38]. In this paper, we use 1.25 t. In China, the price of 1 t of alumina ranges from CNY 2750 to 2850 [40], and in this paper, we use CNY 2800. According to the procurement list from the cooperative cement plant, the purchase price of red mud is CNY 120/t. Combining the above information and applying Equations (3) and (4), we can calculate that the mass allocation coefficient for red mud is approximately 0.556, and the economic allocation coefficient is approximately 0.051.

Next, we consider fly ash and slag, both of which originate from the burning of coal in thermal power generation. According to the literature, burning 1 t of standard coal produces 138 kg of fly ash and approximately 300 kg of slag. For every 1 kWh of electricity generated, 300 g of standard coal is consumed, resulting in the production of about 41 g of fly ash and 90 g of slag [39]. In China, the price of industrial electricity is CNY 0.725/kWh. Based on the cement plant’s procurement list, the current purchase price of fly ash is approximately CNY 30/t, while the purchase price of coal slag is about CNY 15/t [41]. Combining the above information, it becomes evident that fly ash and coal slag are not suitable for mass allocation, as electricity cannot be measured by mass. Therefore, only economic allocation can be adopted. Additionally, since thermal power generation produces multiple by-products, Equation (4) and its derivatives need to be adjusted as follows:

(7) $$\frac{m_{waste\_1}\cdot F_{waste\_1}}{F_{product}+\sum_{i=1}^{n}m_{waste\_i}\cdot F_{waste\_i}}=\frac{m_{waste\_1}\cdot F_{waste\_1}}{F_{upper-summary}}=C_{1}$$

(8) $$C_{m\_1}=\frac{m_{waste\_1}}{1+\sum_{i=1}^{n}m_{waste\_i}}$$

(9) $$C_{e\_1}=\frac{{\$}_{waste\_1}\cdot m_{waste\_1}}{{\$}_{product}+\sum_{i=1}^{n}{\$}_{waste\_i}\cdot m_{waste\_i}}$$

(10) $$F_{cement}=F_{cement-net}+\sum_{i=1}^{n}\frac{m_{waste-process\_i}}{m_{waste\_i}}\cdot F_{upper-summary}\cdot C_{i}+\sum_{i=1}^{n}m_{waste-process\_i}\cdot F_{secondary-working\_i}$$

According to Equation (9), the economic allocation coefficient for fly ash can be calculated as approximately 0.0017, while the coefficient for coal-fired slag is 0.0019. Regarding domestic waste, due to its complex upstream processes and failure to meet the requirements of the WFD, it cannot be considered a by-product and can only be assessed for environmental impact using the CO method at present. Considering the above factors, four parallel LCA analysis cases are set up to comprehensively evaluate the most suitable allocation method for the cement industry.

Case 1: No-alternative control group.

Case 2: Red mud: CO method; Fly ash and slag: CO method; Domestic waste: CO method.

Case 3: Red mud: Mass allocation; Fly ash and slag: Economic allocation; Domestic waste: CO method.

Case 4: Red mud: Economic allocation; Fly ash and slag: Economic allocation; Domestic waste: CO method.

The no-alternative control group represents the theoretical proportion of raw materials for cement production without using any solid waste as alternative raw materials or fuels. Specifically, during the raw meal grinding stage, aluminum oxide red mud and fly ash can serve as siliceous and aluminous materials to replace some of the clay or shale. In the clinker calcination stage, domestic waste is utilized as an alternative fuel to substitute for a portion of the coal. However, in the cement grinding stage, the combination of slag and dust, which enhances cement durability, is not considered as an alternative raw material [42,43]. In summary, for the no-alternative control group, red mud and fly ash are replaced with 126.133 kg of clay, and based on equivalent calorific value, domestic waste is replaced with 20.511 kg of coal. The calorific value data originate from the testing of incoming fuels conducted by the collaborating cement plant.

## 3. Results

Life cycle impact assessment involves the classification and characterization of the environmental impacts caused by the product’s entire life cycle, based on the input and output data aggregated from the life cycle inventory analysis. There are various methods for classifying and characterizing environmental impacts, and in this paper, six indexes are selected for environmental impact assessment, as shown in Table 3.

The environmental impacts for each case are calculated separately, as shown in Table 4.

## 4. Discussion

### 4.1. Economic Allocation and Mass Allocation

**Figure 2:**
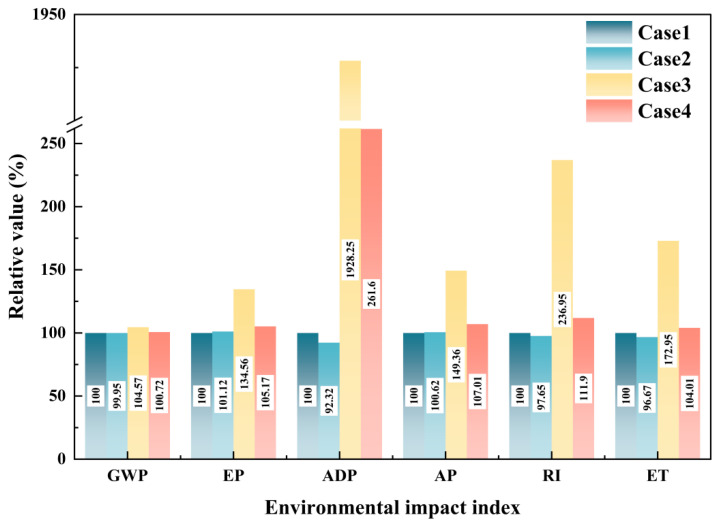
Relative values of environmental impact indexes for each case.

Using Case 1 as the control group, various environmental impact indexes of other cases were processed as percentages, and the results are shown in Figure 2.

According to Figure 2, it can be found that in this series of LCAs, the environmental benefits of using unconventional raw materials such as red mud, fly ash, and domestic waste are limited. Among them, Case 2, which adopts the CO method, has achieved reductions of 0.05%, 7.68%, 2.35%, and 3.33% in GWP, ADP, RI, and ET, respectively, but has not produced environmental benefits in EP and AP. This is a relatively conventional and reasonable result. However, apart from this, the other cases that adopt mass allocation or economic allocation have not produced environmental benefits in any environmental impact indexes. This suggests that some existing energy conservation and emission reduction solutions for cement production are ineffective if strictly following the WFD. This creates a contradictory issue, as using alternative raw materials such as red mud and fly ash as co-disposal processes is a recognized environmental protection effort. The failure of this method at the LCA algorithm level may hinder some cement plants from adopting co-disposal of such solid waste, which is not conducive to reducing the overall environmental impact. Additionally, the environmental impact indexes of Case 3, which adopts the mass allocation method, are higher than those of Case 4 (economic allocation) and Case 2 (CO method). The most prominent difference is observed in ADP, where the result of the mass allocation method is 7.4 times and 20.88 times higher than that of Case 4 and Case 2, respectively. To address these issues, a sensitivity analysis of the inventory can be conducted to identify the most effective improvement points. The sensitivity of raw material inventory in Case 3 is shown in Table 5. The higher the sensitivity, the greater the contribution of this item to overall environmental impact.

**Figure 3:**
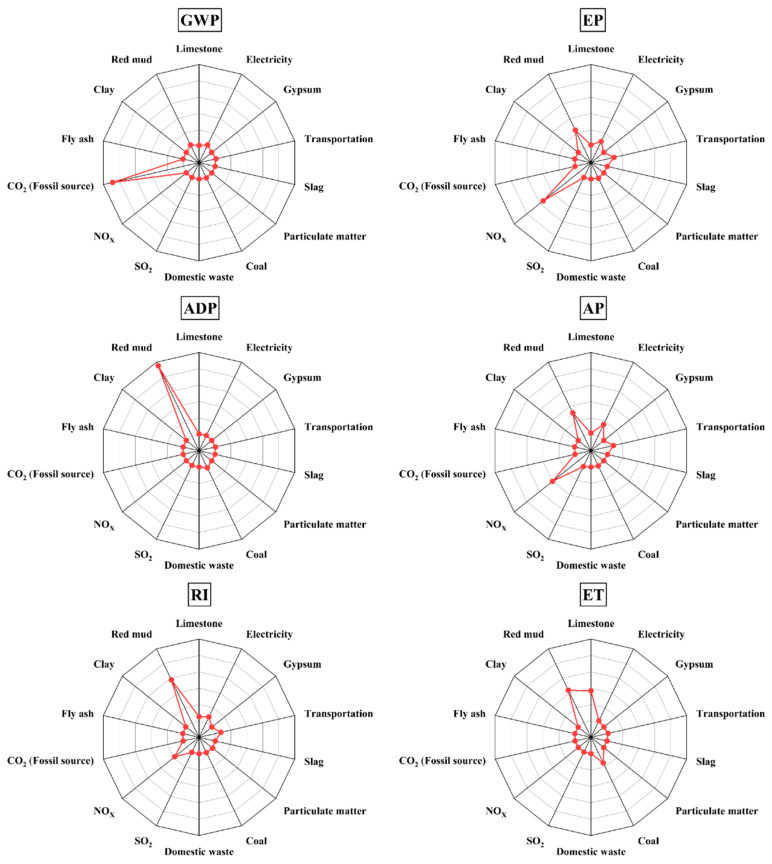
Case 3 inventory sensitivity radar charts.

**Figure 4:**
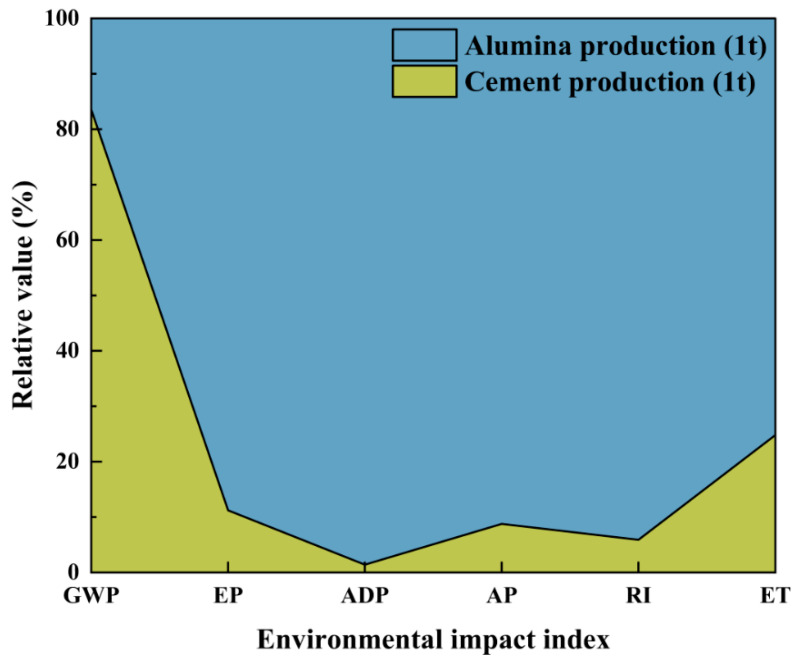
The ratio of the environmental impact of 1 t of cement to the environmental impact of 1 t of alumina (data sourced from CLCD).

Based on Figure 3, it is evident that red mud exhibits high data sensitivity across various environmental impact indexes. For example, in GWP, the contribution of red mud is second only to the direct CO_2_ emissions generated during processes such as raw material decarbonization and fuel combustion in cement production. In EP, AP, and RI, the contribution of red mud is second only to the directly emitted nitrogen oxides. This suggests that red mud may be the reason why Cases 3 and 4 fail to generate environmental benefits even when unconventional raw materials are utilized. Specifically, the sensitivity of red mud in ADP reaches a remarkable 95.16%. According to CML2002 [45], this significant sensitivity can be attributed to the fact that the characterization factor for bauxite in ADP calculations is 2.53 × 10^−5^, whereas the characterization factor for coal is only 3.86 × 10^−8^. This difference is determined by the total proven reserves of minerals on Earth; compared to bauxite, coal, limestone, and clay have huge reserves, resulting in a lower weight in the ADP accounting system. Furthermore, the production of 1 t of alumina typically requires 2.3 t to 2.5 t of bauxite. The combined effect of significant bauxite consumption and thousands of times higher characteristic factors contributes to such a substantial difference. Similarly, as shown in Figure 4, when compared to cement production, the upstream process of red mud, namely alumina production, is a highly polluting and emissive industrial process. Therefore, from the perspectives of mass allocation or economic allocation, the upstream environmental load of by-product red mud is relatively significant.

In addition, it can be seen from Equation (5) that after adopting the allocation method, the environmental load that needs to be accounted for in cement production is directly related to the allocation coefficient *C*. The larger the ratio of *C* to *m_waste_*, the greater the upstream environmental load of solid waste that needs to be calculated. Combining Equations (3) and (4), the following formula can be derived:

(11) $$C_{convert\_m}=\frac{1}{1+m_{waste}}$$

(12) $$C_{convert\_e}=\frac{{\$}_{waste}}{{\$}_{product}+{\$}_{waste}\cdot m_{waste}}$$

(13) $$F_{cement}=F_{cement-net}+m_{waste-process}\cdot F_{upper-summar}\cdot C_{convert}+\;\;m_{waste-process}\cdot F_{secondry-working}$$

where *C_convert_* represents the conversion coefficient, and a larger *C_convert_* indicates a greater upstream environmental load of solid waste that needs to be accounted for.

**Figure 5:**
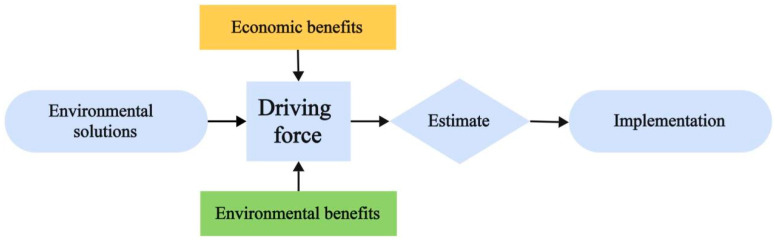
Schematic diagram of the concept of driving force.

Based on the above formulas, the mass *C_convert_* of red mud in this LCA study is calculated to be 0.247, while the economic *C_convert_* is only 0.173 × 10^−4^. Obviously, the economic allocation method allocates a smaller environmental load to downstream processes. From the perspective of market behavior, decision-makers, whether in upstream or downstream processes, must comprehensively consider the pros and cons when making a decision, indicating that they have sufficient “driving force” for this matter, as shown in Figure 5.

Decision-makers typically implement plans after weighing the economic and environmental benefits of unconventional raw materials to generate sufficient driving force. By integrating driving force with the comparison of LCA cases, we can summarize the advantages and disadvantages of economic and mass allocation in cement production.

Mass allocation: The value is stable and determined by the upstream production process, with minimal fluctuations in numerical values. However, it is not suitable for all scenarios. For example, in the upstream process of fly ash production, which involves thermal power generation, the main product, electricity, cannot be measured by mass, and the inherent driving force of economic factors cannot be reflected.

Economic allocation: It has good universality and can fully reflect the comprehensive driving force. However, it is linked to the market price of raw materials and greatly influenced by economic trends, which may shorten the timeliness of the LCA report.

### 4.2. Allocation of Domestic Waste

#### 4.2.1. Active/Passive Environmental Load

In the discussion in 4.1, it is believed that the economic allocation method in cement production can more fully reflect the comprehensive driving force compared to the mass allocation method. However, economic allocation increases the data demand for raw material prices compared to mass allocation. That is, to adopt the economic allocation, apart from satisfying the WFD, the following two data items should also be available:

1. A clear upstream process.

2. The market value of unconventional raw materials and upstream main products.

Obviously, the domestic waste within the system boundary of this LCA does not meet any of the above conditions. Firstly, domestic waste does not have a clear upstream process, as its sources are complex. Moreover, in most cases, domestic waste does not have a traditional market value. Therefore, domestic waste generally cannot be considered a by-product, similar to contaminated soil, etc. To distinguish, this study divides unconventional raw materials in cement production into Valorized Solid Waste (VSW) and Non-Valorized Solid Waste (NVSW). Simply put, VSW refers to solid waste that meets the requirements of the WFD, and the environmental load can be calculated using the economic allocation method. On the other hand, NVSW does not meet the WFD or has no economic value, making it impossible to adopt the economic allocation method.

Due to the uniqueness of its production process, cement production is often regarded as a component of co-disposal technology. Therefore, there is a large amount of solid waste with no economic value as raw materials.

The main reason why these solid wastes have no economic value is that they have no obvious utilization benefits and may even be harmful to cement production. For example, it can lead to an increase in heavy metal content in cement and the formation of crust in cement kilns. Therefore, the application of NVSW often requires the development of new production processes to address these challenges. In other words, overall, using NVSW as raw material is not a rational decision. The CO method can not reflect sufficient driving force. In fact, taking China as an example, cement plants generally engage in such practices motivated by policy guidance and government subsidies. For example, in the cooperative cement plant, the government provides an incentive of CNY 110/t for completing the co-disposal of domestic waste. Compared to the cost of most VSW, this is a considerable revenue. Therefore, it is necessary to design a novel allocation method that comprehensively considers driving force from various aspects.

Before designing a new allocation method, the environmental load of solid waste that needs to be accounted for in LCA research can be divided into four parts, as given below.

**Figure 6:**
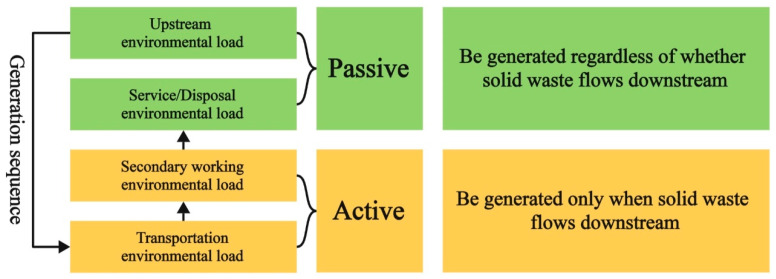
Schematic diagram of the environmental load of solid waste.

As shown in Figure 6, for solid waste, the environmental load that needs to be accounted for includes the upstream environmental load, service/disposal environmental load, secondary working environmental load, and transportation environmental load. Among them, the service/disposal environmental load refers to the direct environmental load generated by the combustion and decomposition of solid waste in cement production. The secondary working environmental load refers to the environmental load of secondary processes such as grinding and drying after the solid waste enters the cement plant. In addition, both the upstream environmental load and the service/disposal environmental load will be generated regardless of whether the solid waste flows to the downstream process. This is because even if the solid waste does not participate in any downstream production process, it still needs to be disposed of eventually, which is unavoidable and occurs before downstream decision-making. Therefore, they are classified as passive environmental loads. On the other hand, the secondary working environmental load and transportation environmental load are generated only when the solid waste flows to the downstream process, which occurs after downstream decision-making. So, they are classified as active environmental loads.

#### 4.2.2. Collaborative Disposal Allocation Method

After clarifying the concepts of active and passive environmental load, further analysis was conducted on the CO method. The results indicate that the CO method actually only excludes the upstream environmental load of solid waste, while the remaining environmental load such as service/disposal environmental load is considered in cement production. This results in cement production taking into account some passive environmental load. However, the passive environmental load arises before downstream decision-making, and therefore, the responsibility should belong to the upstream process. If the passive environmental load is included in the LCA of cement production, it is equivalent to taking on the environmental responsibility of disposing of solid waste for upstream processes to a certain extent, which is unfair. Additionally, for certain solid waste used as alternative fuels, such as domestic waste, their service/disposal process involves combustion, which inherently generates higher emissions. If the service/disposal environmental load is accounted for in the LCA of cement production, it will significantly limit the environmental benefits of using domestic waste as alternative fuel and cannot explain the driving force behind this decision.

Meanwhile, in previous studies, Roozbeh introduced an algorithm that treats some solid waste used in the cement production process as having zero environmental load. This method considers the cement plant’s adoption of solid waste as a disposal process. Not only does it exclude the upstream environmental load of solid waste, but it also excludes the environmental load of transportation, secondary working, and service/disposal. In other words, both active and passive environmental load are accounted for in the upstream process [48], that is,

(14) $$m_{product}\cdot F_{product}=m_{product}\cdot F_{upper-summary}+m_{waste}\cdot F_{disposal}+\;m_{waste}\cdot F_{secondary-working}$$

*F_disposal_* represents the direct environmental load generated by disposing of 1 unit of waste. If the disposal process occurs during downstream processes such as cement production, the environmental load of cement should be calculated as follows:

(15) $$m_{cement}\cdot F_{cement}=m_{cement}\cdot F_{cement-net}-m_{waste-process}\cdot F_{disposal}$$

However, this algorithm is relatively radical and may lead to overly optimistic LCA results for cement production. Additionally, including the active environmental load in the upstream process adds additional environmental load to the upstream process, as the active environmental load occurs after downstream decision-making. This could potentially reduce the incentive for the upstream process to make the decision to send solid waste to cement plants for co-disposal. Furthermore, it establishes a connection between the accounting of upstream and downstream processes, affecting the efficiency of LCA research in various industries. In addition, completely separating the environmental load between upstream and downstream processes can be challenging for data collection related to actual production. For example, considering domestic waste, the algorithm requires separating the combustion emissions from domestic waste and coal. Although this can be achieved with the help of laboratories or databases, implementing it in actual production is challenging, because it is often difficult for pollution monitoring systems to specifically detect pollutants produced by specific fuels.

Simultaneously, Roozbeh’s algorithm offers many valuable insights. For example, it deeply reflects the “polluter pays principle” [49,50,51,52], where the generator of solid waste is responsible for its environmental load; this is a relatively fair method. According to the driving force and active/passive environmental load theory, the passive environmental load should be accounted for in the upstream process of solid waste, while the active environmental load should be included in the downstream process. However, similar to Roozbeh’s algorithm, there is also a challenge in practice: it is difficult to individually detect the service/disposal environmental load of a specific raw material. Based on these issues, for NVSW, a new allocation method can be designed: the Collaborative Disposal Allocation Method (CD method).

(16) $$m_{product}\cdot F_{product}=m_{product}\cdot F_{upper-summary}+m_{waste}\cdot F_{ord-disposal}$$

(17) $$m_{cement}\cdot F_{cement}=m_{cement}\cdot F_{cement-net}+m_{waste-process}\cdot F_{secondary-working}-\;m_{waste-process}\cdot F_{ord-disposal}$$

**Figure 7:**
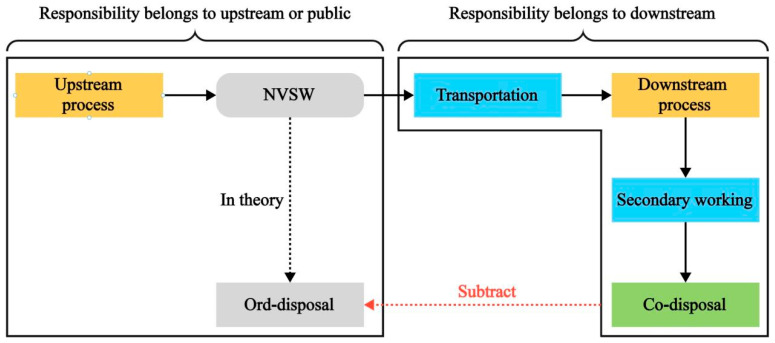
Logical diagram of environmental responsibility attribution in CD method.

*F_ord-disposal_* represents the theoretical environmental load generated by disposing of 1 unit of solid waste through ordinary methods such as incineration or landfill, which is determined by local circumstances. Based on Equations (16) and (17), the accounting of upstream and downstream processes can be completely separated. The downstream process does not account for any environmental load from the upstream process of NVSW, except for the active environmental load incurred after downstream decision-making, such as secondary working and transportation environmental load. Additionally, the service/disposal environmental load of NVSW also needs to be included in downstream process. The theoretical environmental load of ordinary disposal avoided through co-disposal needs to be subtracted from downstream process accounting. This subtracted portion should be accounted for in the upstream process, ensuring that the total social environmental load remains unchanged, aligning with the “polluter pays principle”. If the environmental load of ordinary disposal exceeds that of co-disposal conducted by the downstream process, the difference is considered an environmental benefit for the downstream process. Conversely, if the environmental load of ordinary disposal is less than or equal to that of co-disposal, economic compensation should be provided to the downstream process outside of the LCA accounting, such as economic incentives or tax relief offered by China to cement plants that dispose of domestic waste. Overall, it is essential to ensure that the downstream processes have sufficient driving force to carry out co-disposal efforts. The basic logic of the CD method is shown in Figure 7.

Next, the CD method is adopted to recalculate the environmental impact of 1 t of P·O 42.5 cement. In this process, the ordinary disposal method for domestic waste is sanitary landfill, which is determined by the actual situation of the cement plant’s location. The city’s landfill site for domestic waste is located 25 km away from the sampled cement plant. The calculation results are shown in Table 6 below.

**Figure 8:**
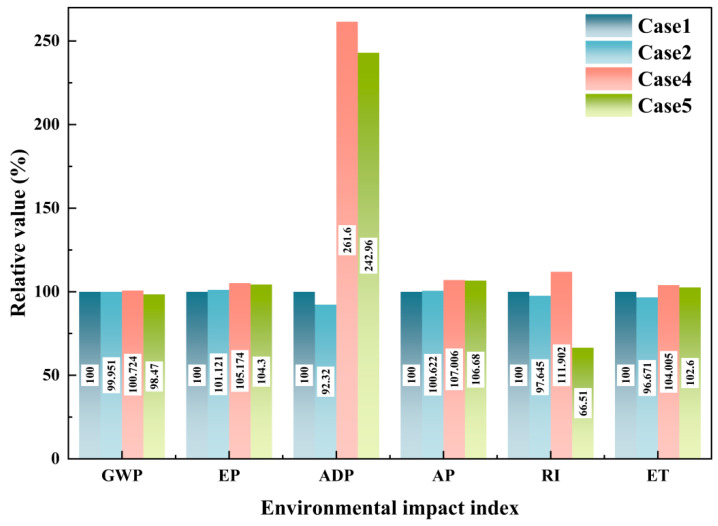
Relative values of environmental impact indexes for each case.

The relative values of environmental impact indexes for Case 2, Case 4, and Case 5 compared to Case 1 are shown in Figure 8.

From Figure 8, it can be observed that, after the adoption of the CD method, there is a noticeable reduction in various environmental impact indexes for the cement plant’s co-disposal of domestic waste compared to Case 4. Specifically, even when incorporating red mud and accounting for its upstream environmental load using economic allocation, Case 5 still shows significant environmental benefits of 1.53% and 33.49% in GWP and RI, respectively, compared to the control group. This notable improvement could be attributed to the substantial transportation of sand and gravel, as well as the open-air construction activities, typically required for the landfill disposal of domestic waste.

## 5. Conclusions

According to the Waste Framework Directive and their roles in production activities, the raw materials used in cement production can be divided into conventional raw materials, Valorized Solid Waste (VSW), and Non-Valorized Solid Waste (NVSW). Based on the actual production data from a typical cement plant located in South China, we conducted a series of LCA case studies employing different allocation methods. We also introduced the concepts of driving force, as well as active and passive environmental load. Through a comparative analysis of five LCA cases, we reasonably inferred the following relatively representative conclusions:

The selection of allocation methods may have a significant impact on the results of LCA for cement production. In some cases, it even directly affects whether the scheme can generate theoretical environmental benefits. When conducting LCA studies, it is essential to choose the most appropriate allocation method based on actual production, industry norms, and legal regulations to enhance the reliability of the LCA research.

For VSW in cement production, the economic allocation method has a higher universality. Meanwhile, compared to the mass allocation method, it comprehensively considers economic factors, thereby more accurately reflecting the decision-making driving force behind the adoption of certain VSW. However, NVSW does not meet the conditions for economic allocation. Therefore, combining the concepts of driving force and active/passive environmental load, and based on the “polluter pays principle”, the Collaborative Disposal Allocation Method (CD method) is proposed. This method can more specifically highlight the specific environmental benefits brought by the co-disposal of solid waste in cement plants and comprehensively reflect the driving force behind decision-making, such as economic benefits and policy orientations.

In the LCA studies of cement production, the choice of allocation methods can significantly influence the final results. However, it is important to note that allocation is a prerequisite for these methods. Regardless of what allocation method is used, the total environmental load on society remains unchanged. The allocation methods are essentially just a division of responsibilities, and cannot generate environmental benefits themselves. And allocation methods can also serve as a tool. Reasonable utilization of allocation methods can encourage enterprises to participate more in the co-disposal of solid waste, thereby promoting the entire society’s environmental load towards a lower direction.

Finally, as described in the text regarding allocation methods, allocation methods are significantly influenced by factors such as industry, location, and regulations. Similarly, LCA studies also exhibit relatively strong industry-specific and regional differences. This leads to difficulties in achieving true unification of LCA algorithms and standards. Although the LCA cases in this paper are based on actual cement production, it only focuses on one cement plant, so the results have relatively weak representativeness in the region and industry. Therefore, this study is more suitable as a methodological reference rather than for a direct replication of its quantitative analyses. To truly achieve standardization of LCA in the cement industry, a broader range of samples and research practices is needed, and this is an ongoing process of continuous improvement.

## Figures and Tables

**Table 1:** Life cycle inventory of P·O 42.5 cement.

Cement (1 t)		Clinker (1 t)		Raw Meal (1 t)	
Clinker	767.760	Raw meal	1540.000	Limestone	798.860
Electricity (kWh)	27.605	Electricity (kWh)	27.713	Electricity (kWh)	13.534
Gypsum	45.649	Coal	136.589	Fly ash	58.903
Limestone	74.036	Domestic waste	38.291	Clay	127.544
Dust	2.766			Red mud	67.230
Slag	141.966				

Note: Except for electricity, all units are in kg.

**Table 2:** Modes of transportation and distances.

Raw Material	Road Transportation (km)	Railway Transportation (km)
Limestone	7.2	0
Fly ash	16	0
Clay	7.2	0
Red mud	199	0
Coal	23	3044.9
Domestic waste	17	0
Gypsum	16	0
By-pass system dust	0	0
Slag	16	0

Note: Road transportation is calculated using 8 t diesel trucks, and select industry average values for railway transportation data. The energy consumption for grinding and belt conveyor transportation after the raw materials enter the factory is already included in the electricity consumption of each section. The by-pass system dust is waste generated from the burning of domestic waste, without any transportation process. It accounts for less than 1% of upstream low-value waste materials and without considering any environmental load.

**Table 3:** Environmental impact indexes.

Index	Units	List Substances	Methods
Global warming potential (GWP)	kg CO_2_ eq ^①^.	CO_2_, CH_4_, N_2_O…	IPCC2013 [44]
Eutrophication potential (EP)	kg PO_4_^3−^ eq.	NH _3_, NH_4_-N, COD…	CML2002 [45]
Abiotic depletion potential (ADP)	kg Sb eq.	Iron, manganese, copper…	CML2002
Acidification potential (AP)	kg SO_2_ eq.	SO_2_, NO_x_, NH_3_…	CML2002
Respiratory inorganics (RIs)	kg PM2.5 eq.	CO, PM_10_, PM_2.5_…	IMPACT2002+ [46]
Ecological toxicity (ET)	CTU_e_	Organic, metal compounds…	USEtox [47]

Note: ① “eq” is the abbreviation of “equivalent”, which refers to the equivalent weight or measure. For example, in GWP, CO_2_ is used as the reference substance, and various other greenhouse gases have their respective CO_2_ equivalent factors based on their greenhouse effect potency. Thus, the emissions of various greenhouse gases during the product’s life cycle can be multiplied by their respective equivalent factors and then summed up to obtain the total GWP, with the unit of kg CO_2_ eq. The same principle applies to ADP, AP, RI, etc.

**Table 4:** Calculation results using different allocation methods.

Label	GWP	EP	ADP	AP	RI	ET
Case 1	6.855 × 10^2^	6.133 × 10^−2^	2.736 × 10^−4^	4.057 × 10^−1^	1.032 × 10^−1^	13.080
Case 2	6.851 × 10^2^	6.201 × 10^−2^	2.526 × 10^−4^	4.082 × 10^−1^	1.007 × 10^−1^	12.644
Case 3	7.168 × 10^2^	8.252 × 10^−2^	52.760 × 10^−4^	6.060 × 10^−1^	2.444 × 10^−1^	22.621
Case 4	6.904 × 10^2^	6.450 × 10^−2^	7.158 × 10^−4^	4.341 × 10^−1^	1.154 × 10^−1^	13.603

**Table 5:** Sensitivity/Contribution rate of Case 3 inventory.

Inventory Item	GWP	EP	ADP	AP	RI	ET
Limestone	1.12%	1.58%	0.38%	1.43%	5.20%	36.74%
Electricity	4.06%	8.89%	0.55%	15.55%	7.58%	2.36%
Gypsum	0.02%	0.04%	0.02%	0.03%	0.05%	0.24%
Transportation	1.19%	8.94%	0.57%	8.23%	7.38%	1.58%
Slag	0.19%	0.41%	0.03%	0.72%	0.35%	0.11%
PM (Particulate matter)	0.00%	0.00%	0.00%	0.00%	1.20%	0.00%
Coal	0.70%	1.02%	3.20%	0.64%	0.71%	14.88%
Domestic waste	0.00%	0.00%	0.00%	0.00%	0.00%	0.00%
SO_2_	0.00%	0.00%	0.00%	1.39%	0.27%	0.00%
NO_X_	0.00%	54.67%	0.00%	40.09%	18.03%	0.00%
CO_2_ (Fossil source)	88.43%	0.00%	0.00%	0.00%	0.00%	0.00%
Fly ash	0.18%	0.40%	0.03%	0.69%	0.34%	0.11%
Clay	0.06%	0.01%	0.07%	0.01%	0.80%	0.09%
Red mud	4.05%	24.04%	95.16%	31.22%	58.10%	43.89%

**Table 6:** Case 5: Red mud: economic allocation; fly ash and slag: economic allocation; domestic waste: CD method.

**Lable**	**GWP**	**EP**	**ADP**	**AP**	**RI**	**ET**
Case 5	6.750 × 10^2^	6.396 × 10^−2^	6.648 × 10^−4^	4.328 × 10^−1^	0.686 × 10^−1^	13.419

## Data Availability

The original contributions presented in this study are included in the article. Further inquiries can be directed to the corresponding author.
